# Toward new perspectives on the interaction of iron and sulfur metabolism in plants

**DOI:** 10.3389/fpls.2013.00357

**Published:** 2013-10-02

**Authors:** Ilaria Forieri, Markus Wirtz, Rüdiger Hell

**Affiliations:** ^1^Department of Molecular Biology of Plants, Centre for Organismal Studies Heidelberg, University of HeidelbergHeidelberg, Germany; ^2^The Hartmut Hoffmann-Berling International Graduate School of Molecular and Cellular Biology, University of HeidelbergHeidelberg, Germany

**Keywords:** iron–sulfur clusters, nutrient sensing, transceptor, sulfate transporter, iron transport

## Abstract

The deficiency of nutrients has been extensively investigated because of its impact on plant growth and yield. So far, the effects of a combined nutrient limitation have rarely been analyzed, although such situations are likely to occur in agroecosystems. Iron (Fe) is a prerequisite for many essential cellular functions. Its availability is easily becoming limiting for plant growth and thus higher plants have evolved different strategies to cope with Fe deficiency. Sulfur (S) is an essential macro-nutrient and the responses triggered by shortage situations have been well characterized. The interaction between these two nutrients is less investigated but might be of particular importance because most of the metabolically active Fe is bound to S in Fe–S clusters. The biosynthesis of Fe–S clusters requires the provision of reduced S and chelated Fe in a defined stoichiometric ratio, strongly suggesting coordination between the metabolisms of the two nutrients. Here the available information on interactions between Fe and S nutritional status is evaluated. Experiments with *Arabidopsis thaliana* and crop plants indicate a co-regulation and point to a possible role of Fe–S cluster synthesis or abundance in the Fe/S network.

## INTRODUCTION

Iron (Fe) is an essential nutrient for plants because of its role as cofactor of many proteins (Balk and Pilon, 2011). Its availability strongly affects plant growth and yield. Thus complex regulatory networks have evolved to tightly balance its uptake, transport, metabolization, and storage during changes of supply and demand. Most of the Fe in plant cells is required in the chloroplasts and in the mitochondria. Several proteins that belong to the electron transport chains in both cellular compartments contain Fe as cofactor, mainly conjugated with sulfur (S) to form the Fe–S clusters. Thus, the biosynthesis of these clusters requires simultaneously reduced S in form of cysteine and of chelated Fe. The provision of the substrates must be tightly regulated for two reasons: (1) in order to meet the plant’s changing demands for Fe–S clusters and (2) to avoid potentially toxic free Fe and sulfide. The strong requirement for reduced Fe and cysteine in stoichiometric amounts to synthesize Fe–S clusters indicates the development of cross-regulatory mechanisms between the two pathways. Indeed preliminary nutrient deficiency studies suggest such a co-regulation but the signals generated by deficiency of S or Fe that are needed to regulate the other pathway have not been addressed so far (Zuchi et al., 2009; Astolfi et al., 2010, 2012). In this respect, an important difference between both nutrients should be noted that may give rise to different regulatory patterns: The major sinks of Fe are heme and Fe–S clusters. In contrast, only a small amount of reduced S is part of cofactors (like Fe–S clusters) and soluble low molecular weight compounds like glutathione, while the bulk is intrinsically bound in proteins in form of cysteine and methionine. Thus, sulfur deficiency does not only limit synthesis of Fe–S cluster containing proteins (Fe–S proteins) but also translation in general. In chloroplasts, the most abundant Fe–S proteins are ferredoxin, photosystem I (PSI), and cytochrome b_6_f complex. Other Fe–S proteins are present in the stroma, including nitrite reductase, sulfite reductase, and adenosine 5′-phosphosulfate reductase (APR). In mitochondria, major Fe–S proteins are complex I, II, and III of the respiratory chain and aconitase in the citric acid cycle. Thus it seems reasonable to assume that the demand of Fe and S for the biosynthesis of Fe–S clusters in the organelles constitutes a feedback signal that co-ordinates the uptake and reduction of both nutrients. In support of this perspective, retrograde signals have been suggested that regulate the uptake and metabolism of Fe during Fe deficiency (Vigani et al., 2013). Such retrograde feedback signals have also been proposed for the S assimilatory pathway (Hell and Wirtz, 2011; Chan et al., 2013).

## UPTAKE AND REDUCTION STRATEGIES AND REGULATION OF THE PATHWAYS

Although Fe is one of the most abundant elements in earth’s crust, its availability is highly restricted in alkaline and calcareous soil, because of Fe^3^^+^ precipitation. Thus Fe can easily become a major constraint for plant growth and higher plants have developed strategies to regulate its homeostasis (Hindt and Guerinot, 2012). One of the prime targets in this respect is the control of iron acquisition in the root. All dicotyledonous and non-graminaceous monocotyledonous plants mobilize iron via the so-called Strategy I or reduction strategy, which is based on soil acidification to increase Fe solubility and reduction of Fe^3^^+^ to Fe^2^^+^ before the uptake. In *Arabidopsis thaliana*, the first step is catalyzed by members of the *Arabidopsis* H^+^-ATPase family AHA, while the ferric-chelate reductase oxidase FRO2 reduces Fe^3^^+^ prior to the uptake into root epidermal cells by iron-regulated transporter 1 (IRT1), a divalent metal transporter. Roots of Strategy II plants from the Poaceae use the extrusion of phytosiderophores to chelate Fe^3^^+^ and import the resulting complexes (Kobayashi and Nishizawa, 2012). In Strategy I plants, two networks are known to control the regulation of the Fe uptake machinery, based on the transcription factors FIT (FER-like iron deficiency induced) and POPEYE, respectively (Ivanov et al., 2012). The permease in chloroplast 1 (PIC1) has been characterized as plastidic Fe importer in *Arabidopsis* (Duy et al., 2007), whereas the mitochondrial iron transporter MIT1 has been identified in rice (Bashir et al., 2011). In acidic and anaerobic conditions, Fe overload can cause severe damage to plant cells, because free Fe catalyzes the formation of reactive oxygen species (ROS). Thus, the concentration of free Fe ions must be tightly regulated. In this regard, the ferritins in plastids and probably also in mitochondria play a fundamental role in the storage of Fe, in the first case preventing photo-oxidative damage (Briat et al., 2010; Tarantino et al., 2010). In the mitochondria, also frataxin might be involved in the protection against oxidative damage (Busi et al., 2006). However, the bulk of Fe is usually bound as metabolically active Fe in Fe–S clusters. The assembly of Fe–S clusters with their apoproteins involves numerous genes and takes place in plastids, mitochondria, and the cytosol (Balk and Pilon, 2011). Thus, the whole plant and cellular metabolism of Fe is well characterized, while sensing and signaling mechanisms still need to be understood.

Unlike Fe, the macro-nutrient S cannot be reduced by plants in the rhizosphere and must be first taken up in the root plasmalemma in form of sulfate by sulfate transporters (SULTR). The expression of high-affinity SULTR1;1 is highly up-regulated in S deficiency conditions. Further members of the SULTR family mediate its allocation in the leaf cells and import into plastids (Cao et al., 2013) where the assimilation takes place (Takahashi et al., 2011). Following activation the resulting adenosine 5′-phosphosulfate is reduced by APR to sulfite which is further reduced to sulfide. Free sulfide can have strong negative impact on cells because it interacts with free thiols on the surface of proteins, with metals such as copper and iron and inhibits the cytochrome *c* of the respiratory chain (Birke et al., 2012). Cysteine represents the main source of S in a reduced form and is fundamental for the biosynthesis of methionine, Fe–S clusters and the redox compound glutathione. The hetero-oligomeric cysteine synthase complex has been identified as an internal sensor for free sulfide to control the flux toward cysteine. In general, the metabolism of S including uptake and APR expression and activity is believed to be regulated by the availability of sulfate in the soil and the internal demand for reduced sulfur. Different regulators of sulfur metabolism have been identified, but the signal transduction toward supply and demand as well as coordination with other assimilatory pathways are unknown (Takahashi et al., 2011).

## EVIDENCES FOR AN INTERACTION BETWEEN IRON AND SULFUR METABOLISM

The effect of the combined shortage of Fe and S has rarely been subjected to studies but provides first hints for a possible co-regulation of their metabolism and particularly the formation of the Fe–S clusters. In tomato plants S deficiency limits the capacity to cope with Fe shortage, preventing the induction of *FRO1* and reducing the activity of IRT1 (Zuchi et al., 2009). From nutrient starvation experiments with the Strategy II plant barley also a positive correlation between the S nutritional status of the plant and its capability of coping with Fe deficiency emerged. One of the first responses to Fe deficiency in the Poaceae is the extrusion of phytosiderophores in the root rhizosphere in order to chelate and solubilize Fe^3^^+^ (Kobayashi and Nishizawa, 2012). Phytosiderophores are derived from nicotianamine that is synthesized from three molecules of *S*-adenosyl-methionine, thus representing another possible junction between Fe and S metabolism. Under S deficiency conditions the release of phytosiderophores was reduced but when barley plants were re-supplied with sulfate, the release of phytosiderophores was increased (Astolfi et al., 2010, 2012). The impact of Fe deprivation on the sulfur assimilation pathway has been recently investigated in durum wheat (Ciaffi et al., 2013). Here Fe shortage triggered several responses that are associated with S deficiency. The expression of genes encoding high-affinity SULTR was up-regulated in the root, as well as of several genes of the S metabolic pathway. These studies have followed a nutritional approach in crop plants and have focused on the changes that occur in the uptake and assimilation processes, supporting the idea of a co-regulation of Fe and S metabolism.

## EVIDENCES FROM *Arabidopsis thaliana*

The model plant *Arabidopsis* is well investigated with respect to Fe and S metabolism. A double nutrient limitation strategy would allow to identify the target genes of potential co-regulation and to connect them to the so far known signal transduction chains of Fe and S metabolism. However, microarray data comprising systematic combinations of Fe and S starvation regimes are currently not available in public databases.

**FIGURE 1:**
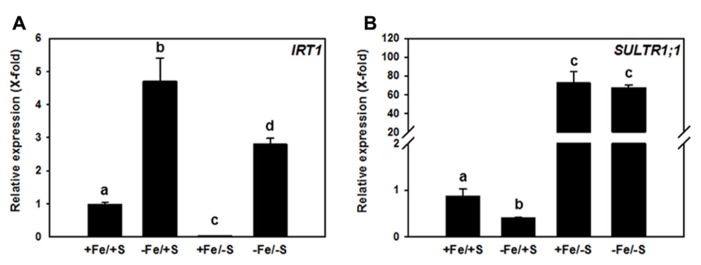
**Relative transcript abundance of *IRT1* (A) and *SULTR1;1***(B)** in roots of 7-week-old *Arabidopsis thaliana* Col-0 plants grown upon different Fe and sulfate supply.** Two weeks old plants were transferred to 6 l boxes containing half strength Hoagland medium with combined Fe and S supply (+Fe = 10 μM FeHBED Iron [*N*, *N*′-di-(2-hydroxybenzyl)-ethylenediamine-*N*,*N*′-diacetic acid], -Fe = 0.1 μM FeHBED, +S = 500 μM MgSO_4_, -S = 1 μM MgSO_4_) and grown for 5 weeks under short day conditions (8.5 h day light, 50% relative humidity, and 0.1 mmol m^-^^2^ s^-^^1^ light intensity). Total RNA was extracted from roots (PeqLAB) and converted into cDNA (Fermentas). The absolute transcript abundance of *IRT1* and *SULTR1;1* was measured by quantitative real-time PCR and normalized against the expression value of the reference gene *TIP41-like* (*At4g34270*). Means ± SD of four replicates are shown. Different letters indicate statistical significance (*P* < 0.05, ANOVA followed by Student–Newman–Keuls test).

The potential impact of a double nutrient shortage approach for Fe and S was tested by growing *Arabidopsis thaliana* plants (Col-0 ecotype) in media that were supplemented with different concentrations of sulfate and of Fe in order to obtain four different growth conditions, named +Fe/+S, -Fe/+S, +Fe/-S, and -Fe/-S. The gene expression of *IRT1* and *SULTR1;1* that are primarily involved in the uptake strategies, was analyzed (**Figure 1**).

The expression of *IRT1* was induced (4.5-fold) in the roots of plants grown in -Fe/+S condition. This expected increase in *IRT1* expression under Fe deficiency was lower when plants were exposed to the double starvation (**Figure 1A**), which might be explained by a decreased requirement of Fe for Fe–S cluster synthesis under -S. In agreement with this, the +Fe/-S treatment caused a 50-fold down-regulation of the expression of the *IRT1* gene from its basal level. Interestingly, Fe deficiency resulted in a 2.5-fold decreased transcript level of the high-affinity SULTR, *SULTR1;1*, when compared to full supply of both nutrients, suggesting that decreased Fe–S cluster synthesis rate and/or accumulation of Fe–S cluster precursors, can affect this sulfate uptake system under -Fe/+S conditions. S depletion induced the expression of *SULTR1;1* by approximately 70-fold irrespective of the Fe supply (**Figure 1B**). This indicates that the demand of cysteine for translation triggers the typical S deficiency response in roots and can overcome the signal generated by decreased demand of cysteine for Fe–S clusters synthesis under -Fe condition.

**FIGURE 2:**
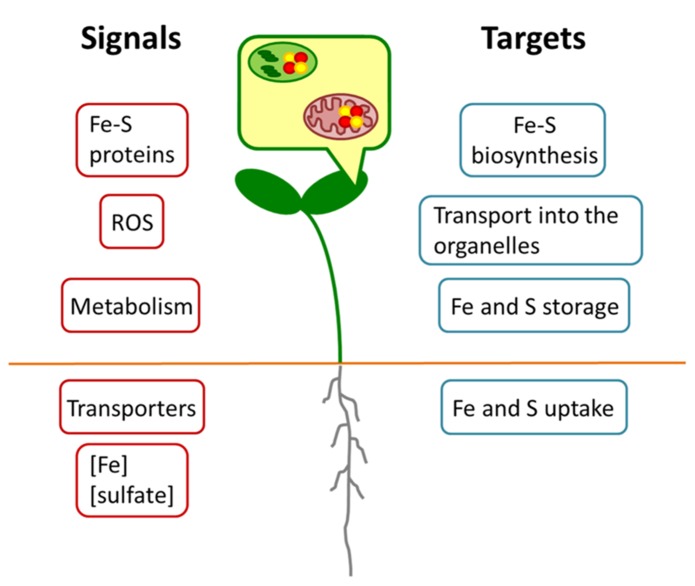
**Schematic model for regulatory interactions between Fe and S metabolism.** Different putative Fe or S signals (red) may originate in the organelles or whole cells of root or shoot. Targets (blue) may comprise metabolic processes at expression or protein levels.

Altogether, these results point toward a co-regulation between the pathways as the limitation of one nutrient influences the uptake of the other one. Such a co-regulation is very likely the outcome of a complex remodeling of the whole plant metabolism upon nutrient limitation as it is known for the prolonged deficiency of the single nutrients (Schuler et al., 2011). Hence, many different signals might contribute to this regulation as outlined in **Figure 2**. A primary signal might come from the sensing of the Fe and S concentrations or even their ratio in either the root environment or the cytosol of root cells. No such sensor has been identified yet, but an intriguing option would be the transporter/receptor (or “transceptor”) concept as described for nitrate uptake (Wang et al., 2012). ROS could be another common signal, since excess of Fe and deficiency of S are known to result in their accumulation (Noctor et al., 2011) and this may also apply to imbalances between the metabolized nutrients. Different studies have indicated that the citric acid cycle is affected upon Fe deficiency (Vigani, 2012). Upon S deprivation, significant changes in the citric acid cycle have also been reported (Nikiforova et al., 2005). Thus, intermediates of the cycle or modulations of energy charge might function as signals for the co-regulation.

The lack of the precursors for the biosynthesis of Fe–S clusters in mitochondria and plastids might constitute an important feedback signal that leads to an overall adjustment of the metabolism. The Fe–S proteins might give rise to a signal for the co-regulation, but when the cluster biosynthesis is impaired, the apoproteins might be degraded and the break-down products might constitute a signal for the metabolic co-regulation. Interestingly, in bacteria Fe–S clusters with transcription factors as apoproteins often serve as sensors, mostly for reactive oxygen (Fleischhacker and Kiley, 2011). The iron regulatory protein 1 (IRP1) that controls the cellular iron homeostasis in mammals and changes between an active cytosolic aconitase with an Fe–S cluster and an apoform that binds iron responsive elements for post-transcriptional regulation is another example of Fe–S cluster sensing but is not present in plants (Arnaud et al., 2007).

All these signals are expected to converge into several plant responses (**Figure 2**). Important targets are the uptake of Fe and S, their transport into organelles, their storage and the actual synthesis of Fe–S clusters. Data mining of earlier microarray analyses of Fe starved *Arabidopsis* (Schuler et al., 2011) pointed to a co-expression cluster of sulfur metabolism-related genes including *APR* and *SULTR* isoforms and the vacuolar sulfate exporters *SULTR4;1* and *SULTR4;2* in leaves, which was interpreted as a necessity of rebalancing the metabolism of S under these conditions (Ivanov et al., 2012). Such a requirement to actively maintain homeostasis of Fe and S metabolism might be the case also for the double nutrient deficiency. It should be taken into consideration, however, that the expression of genes represents only one side of the coin. In Fe and S uptake (e.g., *IRT1*; *SULTR1;1*) numerous post-transcriptional regulatory mechanisms have been observed that can modulate the responses to nutrient deficiency (Takahashi et al., 2011; Hindt and Guerinot, 2012). On the other hand, the enzymatic activity of Fe–S proteins (e.g., APR or aconitase) might be compromised due to the lack of precursors for the biosynthesis of the clusters. As it was already shown by different studies (for overview, see Vigani, 2012), the functionality of several enzymes in the root mitochondria is impaired by Fe starvation. The simultaneous lack of S might exacerbate this effect.

## CONCLUSION

In this perspective the available information on single and combined nutrient limitation with emphasis on *Arabidopsis thaliana* was evaluated and a model of co-regulation between Fe and S metabolism was developed. The model was corroborated by the expression of the two key genes for the uptake of Fe (*IRT1*) and of S (*SULTR1;1*) that correlated with the nutrient supply and was differentially regulated in case of double nutrient deprivation. We conclude that a co-regulation between Fe and S metabolism exists and that Fe–S cluster availability is in accordance with a function in sensing and signaling of combined Fe and S deficiencies.

## Conflict of Interest Statement

The authors declare that the research was conducted in the absence of any commercial or financial relationships that could be construed as a potential conflict of interest.
